# Correlation between liver fibrosis in non-alcoholic fatty liver disease and insulin resistance indicators: a cross-sectional study from NHANES 2017–2020

**DOI:** 10.3389/fendo.2025.1514093

**Published:** 2025-01-31

**Authors:** Bo Yang, Mingsu Gong, Xiaojie Zhu, Yang Luo, Ruiqiu Li, Hai Meng, Yuhan Wang

**Affiliations:** ^1^ Department of Gastroenterology and Hepatology, Guizhou Aerospace Hospital, Zunyi, China; ^2^ Department of Gastroenterology and Hepatology, Binhai County People’s Hospital, Yancheng, China

**Keywords:** non-alcoholic fatty liver disease, liver fibrosis, insulin resistance, logistic regression, TyG-WHtR

## Abstract

**Introduction:**

Non-alcoholic fatty liver disease (NAFLD) is a leading cause of chronic liver disease worldwide, with liver fibrosis (LF) being a crucial pathological feature in the progression of NAFLD. Insulin resistance (IR) is believed to play an important role in the pathogenesis of NAFLD and the development of LF. This study aims to explore the relationship between various IR indicators and LF in patients with NAFLD.

**Methods:**

This study utilized data from the National Health and Nutrition Examination Survey 2017-2020 cycles. Liver steatosis and fibrosis were assessed using liver ultrasound transient elastography. To assess the association between multiple IR indicators and LF, the study methodology included univariate and multivariate logistic regression, as well as restricted cubic spline (RCS) analysis. Subsequently, we used multivariate logistic regression to develop and validate a predictive model for LF, and evaluated the model’s performance using the area under the curve (AUC) and calibration curve.

**Results:**

A total of 904 patients were included in the final analysis. Among these NAFLD patients, 153 (16.92%) had LF. Compared to non-LF patients, LF patients had significantly higher body mass index (BMI), waist circumference (WC), alanine aminotransferase (ALT), aspartate aminotransferase (AST), gamma-glutamyl transferase (GGT), HbA1c, and fasting blood glucose (FBG) levels (all p < 0.05). Analysis of IR indicators showed that LF patients had significantly higher levels of TyG, TyG-WHtR, TyG-BMI, TyG-WC, TyG-GGT, METS-IR, and HOMA-IR (all p < 0.05). After adjusting for covariates, TyG-WHtR remained an independent risk factor (OR=2.69; 95% CI: 2.08-3.47), indicating a strong correlation with LF. The developed nomogram, incorporating AST, TyG, TyG-BMI, and diabetes, showed an AUC of 0.809 (95% CI: 0.771-0.847), indicating good predictive performance for LF in NAFLD patients.

**Conclusions:**

This study confirms that a significant association between various IR and LF in NAFLD patients, and the developed nomogram provides a practical tool for early risk assessment. These findings underscore the clinical value of incorporating IR indices into routine practice to identify high-risk patients, enabling timely interventions to prevent fibrosis progression and improve outcomes.

## 1 Introduction

Non-alcoholic fatty liver disease (NAFLD) is defined as the excessive accumulation of fat in the liver in the absence of significant alcohol consumption. It is often regarded as the hepatic manifestation of metabolic syndrome and is commonly associated with metabolic disorders such as obesity, type 2 diabetes, and hyperlipidemia (1). In recent years, the incidence of NAFLD has increased, surpassing that of viral hepatitis to become the predominant chronic liver disease globally (2). The pathogenesis of NAFLD is complex and ranges from simple fatty liver, characterized by excess fat in the liver without significant inflammation or fibrosis, to non-alcoholic steatohepatitis (NASH), which not only involves fat accumulation but also accompanies liver cell inflammation and damage, ultimately leading to liver fibrosis (LF) (3). LF is a key pathological feature in the progression of NAFLD and a major risk factor for the development of cirrhosis and hepatocellular carcinoma. In recent years, an increasing number of studies have focused on the epidemiology of LF caused by NAFLD, with results indicating that the prevalence of LF significantly increases with the severity of NAFLD (4, 5). Therefore, it is crucial to promptly identify the risk factors for LF in patients with NAFLD.

Insulin resistance (IR) is a well-recognized factor in the pathogenesis of NAFLD and plays a critical role in its progression. IR leads to an imbalance in lipid metabolism, promoting hepatic fat accumulation and contributing to liver inflammation and fibrosis (6). Given the close relationship between IR and NAFLD, indicators of IR, such as fasting blood glucose (FBG), fasting insulin, and the homeostasis model assessment of insulin resistance (HOMA-IR), have been widely used as biomarkers to assess metabolic dysfunction in patients with NAFLD (7, 8). In addition to traditional markers of IR, the triglyceride-glucose index (TyG) has drawn increasing attention in recent years. The TyG index is a calculated measure based on fasting triglycerides and FBG. Due to its simplicity, ease of availability, and strong correlation with IR, it has been widely utilized for assessing IR and cardiovascular disease risk (9, 10). Moreover, the indicators combining TyG with body mass index (BMI), waist circumference (WC), and waist-to-height ratio (WHtR) further enhance the assessment of an individual’s metabolic status and have been shown to be closely associated with the presence and severity of NAFLD (11). Although the association between NAFLD and IR is well-established, the exact relationship between various IR indicators (including the TyG index) and the degree of LF in NAFLD remains unclear. Most previous studies have primarily focused on the presence of NAFLD and its progression to NASH, with comparatively less attention given to the specific correlation between these IR indicators and the degree of hepatic fibrosis in NAFLD patients (12, 13). A deeper exploration of the association between the TyG index and HOMA-IR with LF in NAFLD patients will enhance our understanding of the metabolic mechanisms underlying the disease and provide new insights for early risk assessment.

Histopathological examination of liver biopsy specimens has long been considered the gold standard for diagnosing NAFLD and LF. Nonetheless, this method presents several limitations, including its invasive nature, low acceptability, and high cost (14). In recent years, liver ultrasound transient elastography (LUTE) has emerged as an accurate and non-invasive technique for assessing the degree of steatosis and fibrosis in patients with NAFLD (15). A meta-analysis found that LUTE exhibits good sensitivity and specificity for LF, with sensitivity and specificity values of 0.79 and 0.78, respectively (16). Previous research has focused on developing non-invasive diagnostic methods for LF. Several studies have developed serological models based on biochemical markers and clinical information to predict LF, including the fibrosis-4 index, aspartate aminotransferase (AST) to platelet ratio, AST to alanine aminotransferase (ALT) ratio, Forns index, and BARD score (17–19). However, when these scoring systems are used to predict LF in NAFLD patients, they do not include metabolic indicators such as the TyG index. The lack of these key metabolic markers may reduce the accuracy of the models, failing to fully reflect the fibrosis risk caused by NAFLD.

In our study, we aim to utilize data from the National Health and Nutrition Examination Survey (NHANES) database to assess NAFLD and LF using LUTE. We will then explore the correlation between LF and various IR indicators in NAFLD patients. Additionally, we will attempt to develop a predictive model for NAFLD-related LF based on these metabolic indicators. This study will provide valuable insights into the potential role of IR in the progression of LF and help identify key markers for early risk stratification and management, thereby enabling a more accurate prediction of fibrosis risk in NAFLD patients.

## 2 Materials and methods

### 2.1 Study design and participants

**Figure 1:**
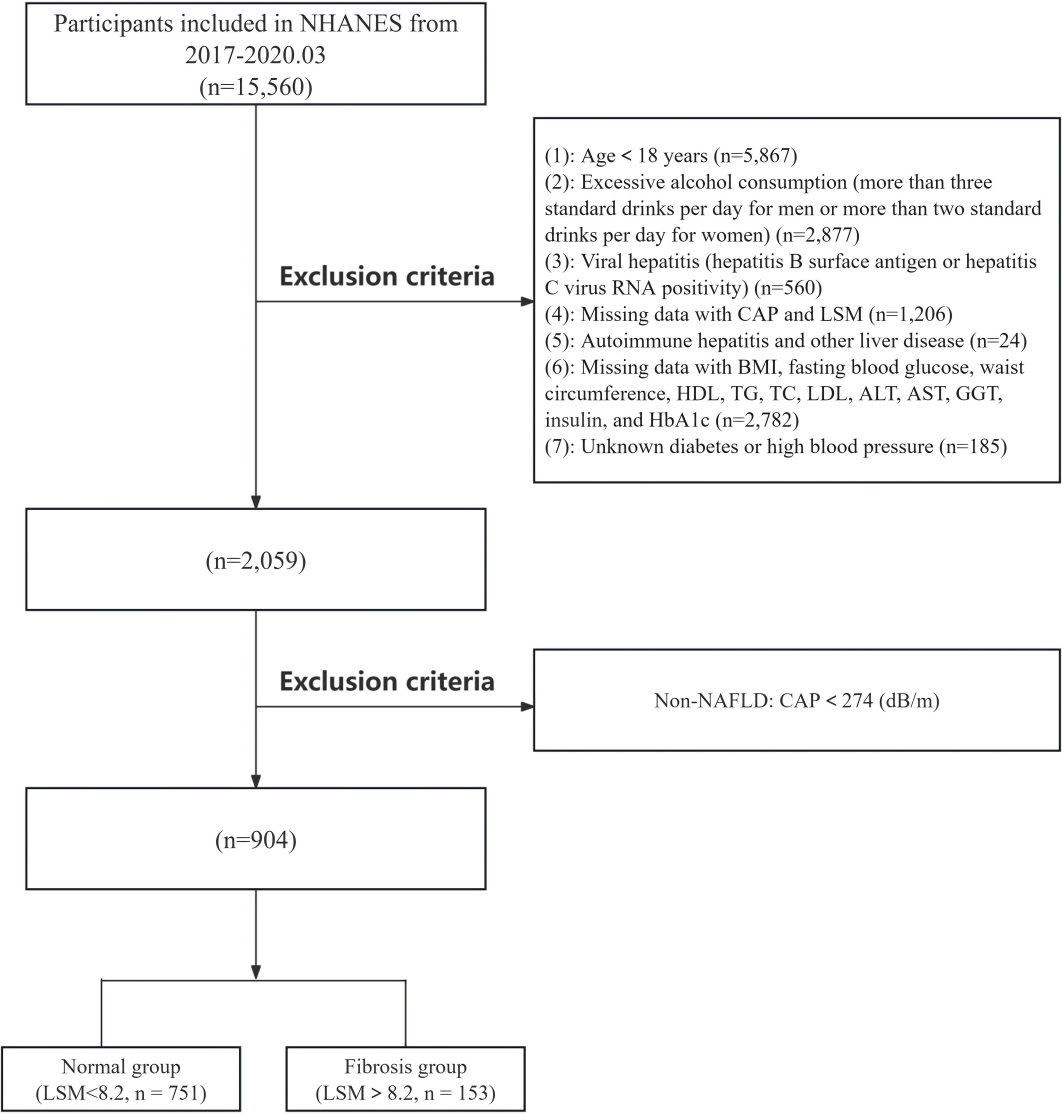
Flowchart of inclusion and exclusion criteria for NAFLD patients in the NHANES database. NAFLD, non-alcoholic fatty liver disease.

The NHANES is a complex, multistage, cross-sectional survey conducted every two years to assess the health and nutritional status of adults in the United States. This study utilized NHANES data from the 2017 to March 2020 cycles, with a total sample size of 15,560 individuals. The following participants were excluded: individuals under 18 years of age (n=5,867), those with excessive alcohol consumption (more than 3 drinks per day for men or more than 2 drinks per day for women, n=2,877), individuals with viral hepatitis (including those positive for hepatitis B surface antigen or hepatitis virus RNA, n=560), individuals with a history of autoimmune hepatitis or other liver diseases (n=24), individuals lacking LUTE data (n=1,206), and individuals missing covariate data (including BMI, FBG, WC, high-density lipoprotein (HDL), triglycerides (TG), total cholesterol (TC), low-density lipoprotein (LDL), ALT, AST, diabetes, and hypertension, n=2,967). Additionally, patients with non-NAFLD (n=1,155) were excluded. In the final analysis, 904 participants with NAFLD were included. A detailed flowchart is shown in **Figure 1**. The specific original data can be found in the **Supplementary Materials**. The NHANES study protocol received approval from the National Center for Health Statistics Research Ethics Review Board, and all participants were fully informed and provided written consent in compliance with the ethical guidelines.

### 2.2 Definition of NAFLD and LF

The definition of NAFLD and LF was primarily determined using LUTE, which provided liver stiffness measurements (LSM), and simultaneously measured the ultrasound attenuation associated with liver steatosis, recorded as the controlled attenuation parameter (CAP). Specifically, CAP≥274 dB/m was used to define NAFLD, and participants with LSM ≥ 8.2 kPa were defined as having LF (20).

### 2.3 Definitions of IR index

The different IR indices were calculated by the following equations (21–25):


WHtR = WC (cm) / height (cm)

TyG = ln [(TG (mg/dL) × FPG (mg/dL))/2]

TyG−WC = TyG × WC

TyG−BMI = TyG × BMI

TyG−WHtR = TyG ×WHtR

TyG−GGT = TyG × GGT

$$\begin{array}{c} \text{METS}-\text{IR}=\text{ ln }\left[\left(2\;\times \text{ FPG }\left(\text{mg}/\text{dL}\right)\right)+\text{ TG }\left(\text{mg}/\text{dL}\right)\right]\\ \;\times \text{ BMI }/\text{ ln }\left(\text{HDL }\left(\text{mg}/\text{dL}\right)\right) \end{array}$$

$$\begin{array}{c} \text{HOMA}-\text{IR }=\;(\text{FPG }\left(\text{mg}/\text{dL}\right)\\ \;\times \text{ fasting insulin }\left(\text{μU}/\text{mL}\right))\;/\;22.5 \end{array}$$

### 2.4 Covariates

In our study, we identified several potential factors associated with LF in NAFLD patients, known as covariates, including variables such as ALT, AST, BMI, and WC, which have been previously reported to be related to the occurrence of LF (26, 27). To control for the influence of these confounding factors on our study results, we implemented covariate adjustments in our statistical models to minimize potential bias. Specifically, our analytical approach included adjustments for the following covariates: demographic characteristics (age, gender, BMI, WC), laboratory indicators (ALT, AST, TC, TG, HDL, LDL, and FBG), and underlying diseases (self-reported physician-diagnosed hypertension or diabetes, and current use of antihypertensive or antidiabetic medications as indicators of hypertension or diabetes). These standardized interviews and questionnaires were administered by trained healthcare professionals.

### 2.5 Statistical analysis

The baseline characteristics of all included patients were stratified based on the occurrence of LF. Non-normally distributed variables were presented as interquartile ranges and compared using the Wilcoxon rank-sum test. Categorical variables were expressed as percentages and compared using the chi-square test. To investigate the relationship between various factors and LF in patients with NAFLD, we initially conducted a univariate logistic regression analysis and visualized the results using a forest plot, which presented the odds ratio (OR) along with their corresponding 95% confidence interval (CI) for each factor. Subsequently, we constructed four multivariate logistic regression models to further assess the independent associations between each IR indicator and LF. The OR and their 95% CI for all models were calculated by exponentiating the regression coefficients, with adjustments for potential confounding factors incorporated in the multivariate models (28). Additionally, the study group used restricted cubic spline (RCS) plots to visualize the linear relationship between IR indicators and LF in NAFLD patients more intuitively (29, 30). The value of IR indicators for diagnosing disease prognosis was assessed using receiver operating characteristic (ROC) curves (31). Based on the multivariate logistic regression models, a nomogram was constructed using statistically significant indicators to diagnose the disease (32). To evaluate the validity of the nomogram, the area under the ROC curve (AUC) and calibration curves were calculated. All statistical analyses were performed using R software (version 4.3.0) and STATA 17.0 (64-bit), with a two-sided P-value <0.05 considered statistically significant.

## 3 Results

### 3.1 Demographic and clinical characteristics of participants

The study cohort included a total of 904 NAFLD patients based on inclusion and exclusion criteria, comprising 751 non-LF patients (83.08%) and 153 LF patients (16.92%). **Table 1** compares the baseline clinical characteristics between patients with LF and those without. Analysis revealed that compared to non-LF patients, LF patients had significantly higher BMI (median: 31.10 [27.90, 35.45] vs. 37.30 [32.50, 43.80], p < 0.001) and WC (median: 106.70 [98.00, 116.50] vs. 122.20 [112.50, 131.70], p < 0.001). Analysis of laboratory markers indicated that LF patients had significantly higher levels of ALT, AST, GGT, HbA1c, and FBG, while TC, HDL, and LDL were significantly lower compared to non-LF patients (all p < 0.05). Analysis of various IR indicators showed that LF patients had significantly higher TyG, TyG-WHtR, TyG-BMI, TyG-WC, TyG-GGT, METS-IR, and HOMA-IR compared to non-LF patients (all p < 0.05). Among patients with comorbidities, those with diabetes or hypertension were significantly more likely to develop LF than those without (all p < 0.05). For other variables, no significant differences were found in sex or age between the two groups (all p > 0.05).

**Table 1:** Baseline demographic and clinical characteristics of participating patients.

Characteristics	Total No. (%)	Non-Fibrosis	Fibrosis	*p*-value
		No. (%)	No. (%)	
Total	904	751 (83.08)	153 (16.92)	
Gender, n(%)				0.322
Male	493 (54.5%)	404 (53.8%)	89 (58.2%)	
Female	411 (45.5%)	347 (46.2%)	64 (41.8%)	
Age (years)	57.00(43.00, 67.00)	57.00(42.00, 67.00)	59.00(47.00, 68.00)	0.220
BMI	32.00(28.30, 37.20)	31.10(27.90, 35.45)	37.30(32.50, 43.80)	<0.001
WC	108.85(99.70, 119.82)	106.70(98.00, 116.50)	122.20(112.50, 131.70)	<0.001
ALT (U/L)	21.00(15.00, 30.00)	20.00(15.00, 28.00)	25.00(16.00, 40.00)	<0.001
AST (U/L)	19.00(16.00, 24.00)	19.00(16.00, 24.00)	21.00(17.00, 29.00)	<0.001
GGT (U/L)	24.00(18.00, 35.00)	24.00(17.00, 33.00)	29.00(21.00, 54.00)	<0.001
TC (mmol/L)	4.63(4.01, 5.38)	4.65(4.09, 5.48)	4.42(3.83, 5.04)	<0.001
TG (mmol/L)	1.29(0.90, 1.79)	1.28(0.89, 1.79)	1.32(0.95, 1.77)	0.592
HDL (mmol/L)	1.16(1.01, 1.40)	1.19(1.01, 1.42)	1.14(0.98, 1.32)	0.044
LDL (mmol/L)	2.74(2.20, 3.14)	2.82(2.25, 3.46)	2.46(1.99, 3.13)	<0.001
HbA1c (%)	5.80(5.50, 6.50)	5.80(5.40, 6.25)	6.20(5.70, 7.60)	<0.001
FBG (mmol/L)	6.11(5.61, 7.11)	6.05(5.55, 6.94)	6.72(5.94, 8.55)	<0.001
TyG	8.77(8.39, 9.23)	8.72(8.37, 9.21)	8.93(8.51, 9.26)	0.005
TyG-WHtR	5.78(5.15, 6.46)	5.60(5.07, 6.23)	6.51(5.90, 7.20)	<0.001
TyG-BMI	282.69(246.32, 333.08)	274.72(241.95, 316.41)	336.98(292.24, 385.13)	<0.001
TyG-WC	961.78(857.68, 1074.51)	939.90(846.10, 1046.47)	1091.97(997.30, 1199.98)	<0.001
TyG-GGT	215.80(154.08, 309.09)	208.09(147.84, 293.14)	266.58(183.50, 502.69)	<0.001
METS-IR	49.57(42.89, 58.82)	48.00(41.88, 56.52)	59.07(51.54, 68.38)	<0.001
HOMA-IR	4.41(2.75, 7.22)	4.05(2.59, 6.15)	7.48(4.29, 11.05)	<0.001
Diabetes, n(%)				<0.001
YES	254 (28.1%)	176 (23.4%)	78 (51.0%)	
NO	650 (71.9%)	575 (76.6%)	75 (49.0%)	
Hypertension, n(%)				<0.001
YES	445 (49.2%)	349 (46.5%)	96 (62.7%)	
NO	459 (50.8%)	402 (53.5%)	57 (37.3%)	

BMI, Body Mass Index; WC, Waist Circumference; ALT, Alanine Aminotransferase; AST, Aspartate Aminotransferase; GGT, Gamma-Glutamyl Transferase; TC, Total Cholesterol; TG, Triglycerides; HDL, High-Density Lipoprotein Cholesterol; LDL, Low-Density Lipoprotein Cholesterol; FBG, Fasting Blood Glucose; TyG, Triglyceride-Glucose Index; TyG-WHtR, Triglyceride-Glucose Index to Waist-to-Height Ratio; TyG-BMI, Triglyceride-Glucose Index to Body Mass Index; TyG-WC, Triglyceride-Glucose Index to Waist Circumference; TyG-GGT, Triglyceride-Glucose Index to Gamma-Glutamyl Transferase; METS-IR, Metabolic Score for Insulin Resistance; HOMA-IR, Homeostasis Model Assessment of Insulin Resistance.

### 3.2 Analysis of factors contributing to LF in NAFLD patients

**Figure 2:**
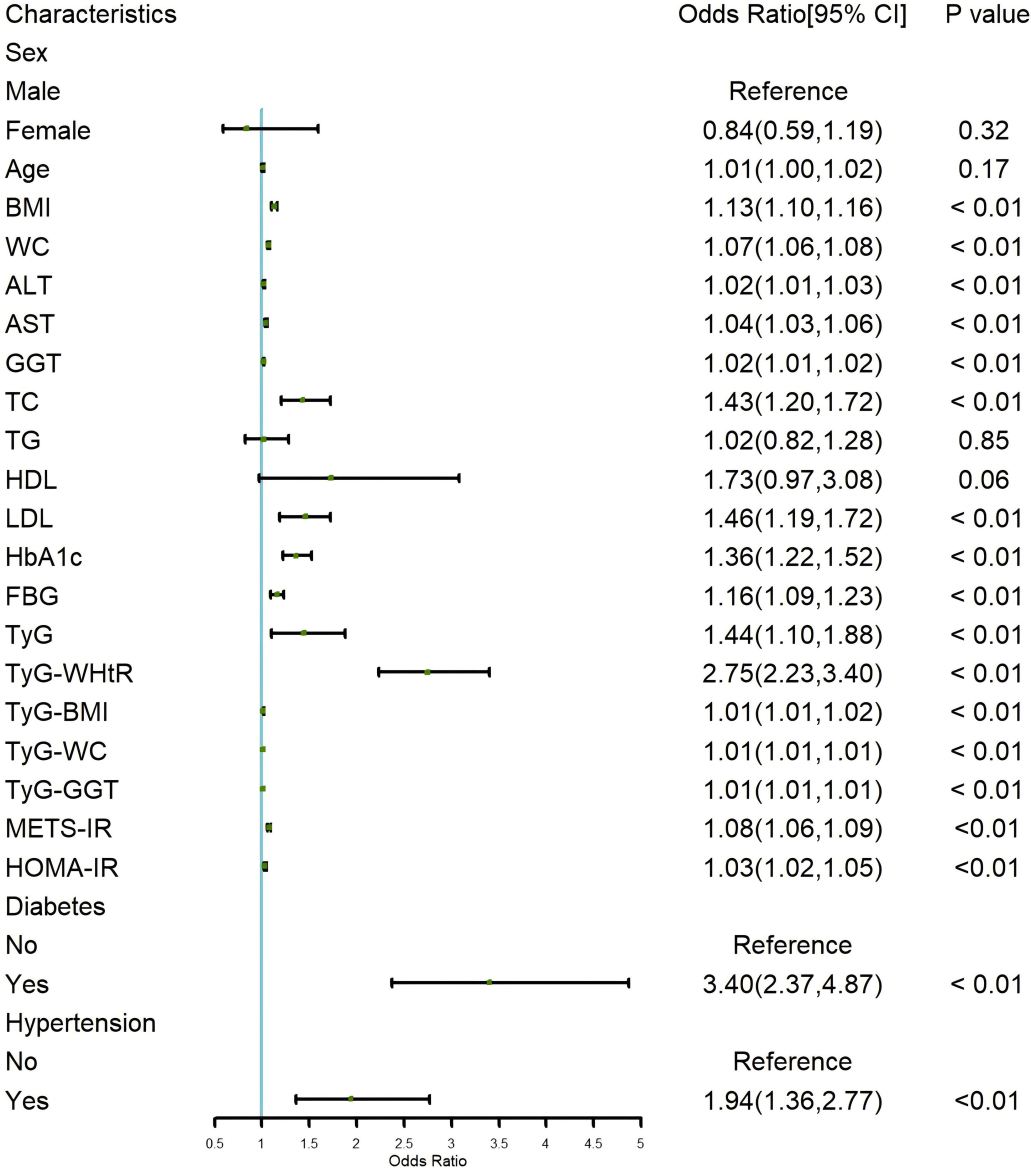
Forest plot of univariate logistic regression for risk factors of LF in NAFLD Patients. LF, liver fibrosis; NAFLD, non-alcoholic fatty liver disease.

To identify the factors associated with the progression of NAFLD to LF, we performed a univariate logistic regression analysis, as shown in the forest plot in **Figure 2**. The analysis revealed that IR indicators, including TyG, TyG-WHtR, TyG-BMI, TyG-WC, TyG-GGT, METS-IR, and HOMA-IR, were significantly associated with the development of LF in NAFLD patients (all p < 0.05). Among these, TyG (OR=1.44; 95% CI: 1.10-1.88, p < 0.01) and TyG-WHtR (OR=2.75; 95% CI: 2.23-3.40, p < 0.01) showed the most notable associations. Additionally, we found that a higher BMI increased the likelihood of LF in NAFLD patients (OR=1.13; 95% CI: 1.10-1.16, p < 0.01). Similarly, higher values of WC, ALT, AST, GGT, TC, LDL, HbA1c, and FBG were associated with an increased risk of LF, with LDL (OR=1.46; 95% CI: 1.19-1.72, p < 0.01) and HbA1c (OR=1.36; 95% CI: 1.22-1.52, p < 0.01) being particularly relevant. Analysis of comorbidities showed that patients with hypertension (OR=3.40; 95% CI: 2.37-4.87, p < 0.01) and diabetes (OR=1.94; 95% CI: 1.36-2.77, p < 0.01) were more likely to develop LF.

**Figure 3:**
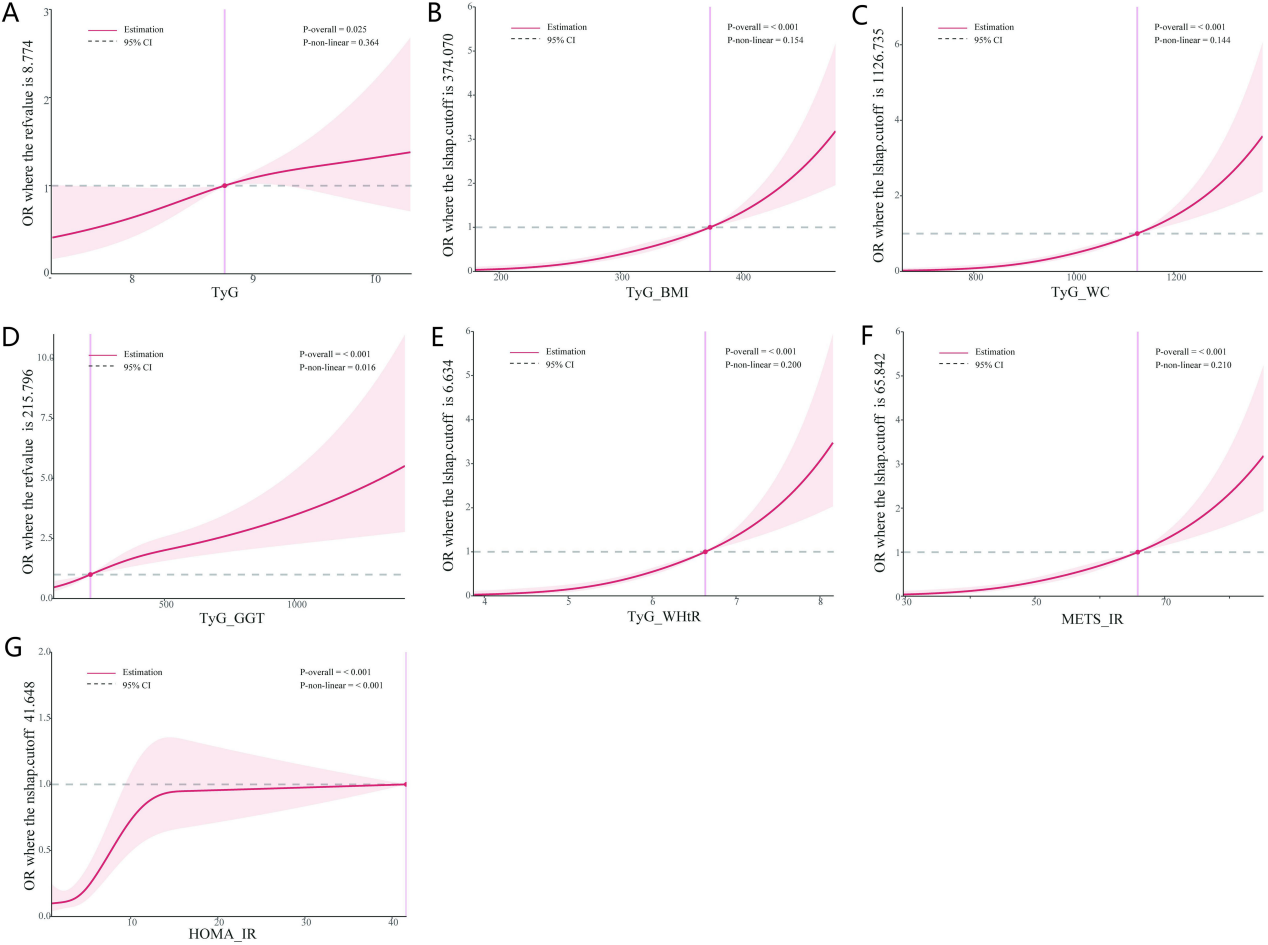
Dose-response between IR indices and the risk of LF. **(A)** Dose-response between TyG and the risk of LF. **(B)** Dose-response between TyG-BMI and the risk of LF. **(C)** Dose-response between TyG-WC and the risk of LF. **(D)** Dose-response between TyG-GGT and the risk of LF. **(E)** Dose-response between TyG-WHtR and the risk of LF. **(F)** Dose-response between METS-IR and the risk of LF. **(G)** Dose-response between HOMA-IR and the risk of LF. IR, insulin resistance; LF, liver fibrosis.

Based on the results of the logistic regression analysis, an RCS plot was constructed to visualize the relationship between different IR indicators and the risk of LF in NAFLD patients (**Figure 3**). It was found that TyG, TyG-WHtR, TyG-BMI, TyG-WC, TyG-GGT, METS-IR, and HOMA-IR were positively correlated with the development of LF in NAFLD patients, further validating the above findings.

### 3.3 Analysis of independent risk factors for LF in NAFLD patients

We constructed four multivariate logistic regression models to further determine whether IR is an independent risk factor for LF in NAFLD patients (**Table 2**). In Model 1, which was unadjusted for any variables, the analysis showed that TyG (OR=1.44; 95% CI: 1.10-1.88, p < 0.01) and TyG-WHtR (OR=2.75; 95% CI: 2.23-3.40, p < 0.01) were most significantly associated with LF, consistent with the univariate logistic regression results, while the other IR indicators were also statistically significant but had weaker associations. After adjusting for age and sex in Model 2, the analysis revealed that the association for TyG-WHtR became more pronounced (OR=3.01; 95% CI: 2.40-3.76, p < 0.01), while the other indicators showed no significant changes compared to Model 1. In Model 3, we further adjusted for comorbidities such as diabetes and hypertension based on Model 2. It was found that the association of TyG-WHtR (OR=2.66; 95% CI: 2.10-3.37, p < 0.01) weakened significantly, and TyG lost statistical significance after adjustment, while the other indicators remained largely unchanged and were all statistically significant (all p < 0.05). Subsequently, in Model 4, additional adjustments for BMI, WC, ALT, AST, GGT, TC, LDL, HbA1c, and FBG were made based on Model 3. It was found that TyG-GGT and METS-IR were no longer statistically significant, while the other indicators remained significantly associated, with TyG (OR=1.23; 95% CI: 1.09-1.45, p = 0.04) and TyG-WHtR (OR=2.69; 95% CI: 2.08-3.47, p < 0.01) being the most notable. Through the construction of these different models, we found that TyG, TyG-WHtR, TyG-BMI, TyG-WC, and HOMA-IR were independent risk factors for the development of LF in NAFLD patients, with strong associations.

**Table 2:** Multivariate logistic regression models assessing IR as an independent risk factor for LF in NAFLD patients.

	Model 1	Model 2	Model 3	Model 4
	OR (95% CI), *p*-value	OR (95% CI), *p*-value	OR (95% CI), *p*-value	OR (95% CI), *p*-value
TyG	1.44(1.10,1.88)< 0.01	1.44(1.09–1.91)< 0.01	1.02(0.75–1.37) 0.91	1.23(1.09–1.45) 0.04
TyG-WHtR	2.75(2.23,3.40)< 0.01	3.01(2.40–3.76)< 0.01	2.66(2.10–3.37)< 0.01	2.69(2.08–3.47)< 0.01
TyG-BMI	1.01(1.01,1.02)< 0.01	1.02(1.01–1.02)< 0.01	1.01(1.01,1.01)< 0.01	1.01(1.01,1.01) 0.02
TyG-WC	1.01(1.01,1.01)< 0.01	1.01(1.01,1.01)< 0.01	1.01(1.01,1.00)< 0.01	1.01(1.01,1.01) 0.02
TyG-GGT	1.01(1.01,1.01)< 0.01	1.01(1.01,1.02)< 0.01	1.01(1.01,1.02)< 0.01	0.99(0.98,1.01) 0.82
METS-IR	1.08(1.06,1.09)< 0.01	1.08(1.06,1.10)< 0.01	1.07(1.06,1.09)< 0.01	0.95(0.90,1.01) 0.06
HOMA-IR	1.03(1.02,1.05)< 0.01	1.03(1.01,1.05)< 0.01	1.02(1.01,1.04)< 0.01	1.01(1.01,1.02)< 0.01

Model 1 was a non-adjusted model.

Model 2 was adjusted for age (years), gender and race.

Model 3 was adjusted for the same parameters as Model 2 with additional adjustments for hypertension (No or Yes) and diabetes (No or Yes).

Model 4 was adjusted for the same parameters as Model 3 with additional adjustments for BMI、WC、ALT、AST、GGT、TC、LDL、HbA1c、FBG.

OR, odds ratio; 95% CI, 95% confidence interval.

IR, insulin resistance; LF, liver fibrosis; NAFLD, non-alcoholic fatty liver disease.

### 3.4 Predictive value of multiple IR indicators for diagnosing LF in NAFLD patients

**Figure 4:**
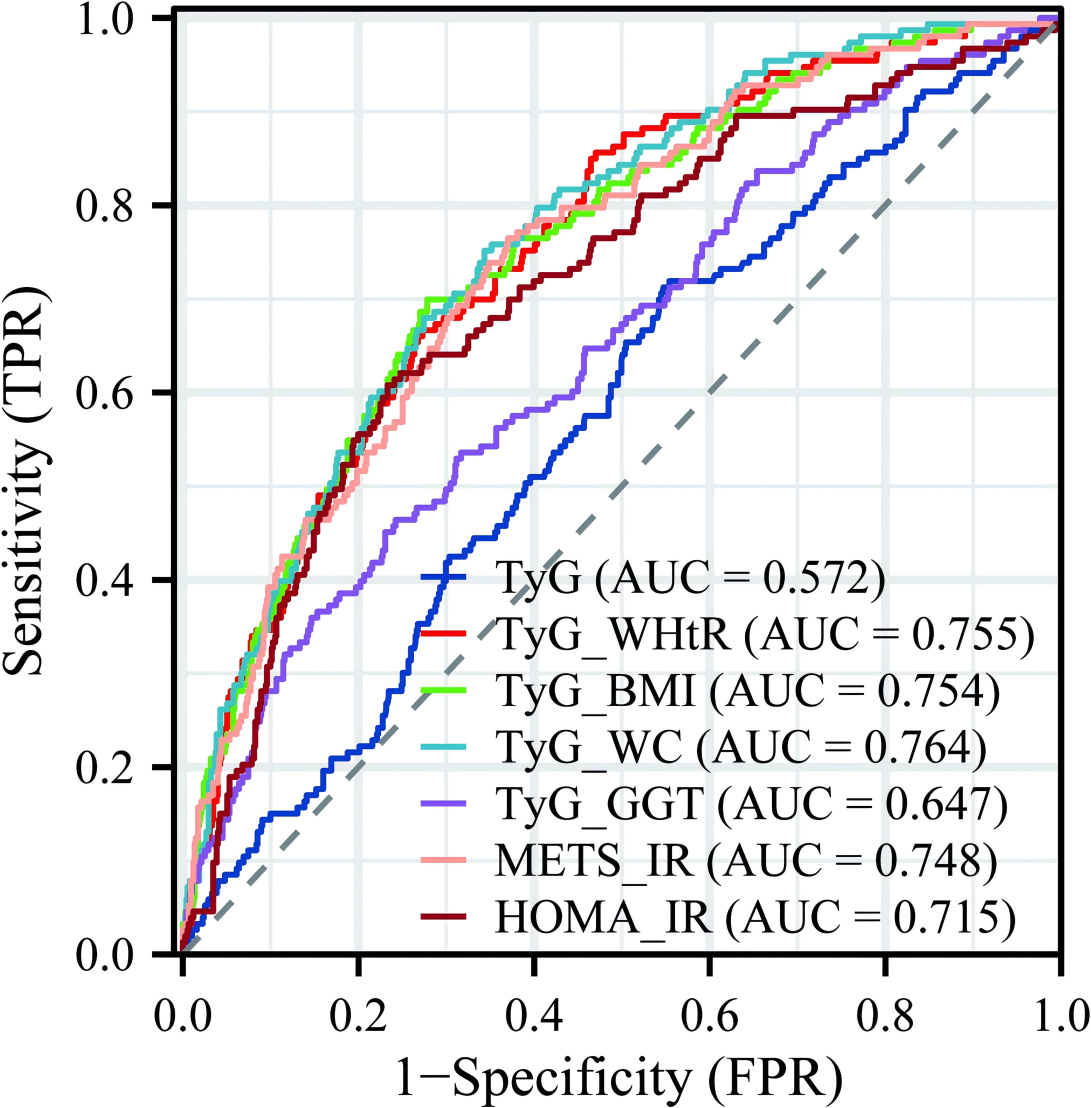
Predictive value of multiple IR indicators for diagnosing LF. IR, insulin resistance; LF, liver fibrosis.

To further explore the clinical diagnostic predictive value of various IR indicators for LF in NAFLD patients, an ROC curve diagnostic analysis model was established (**Figure 4**). The analysis revealed that TyG and TyG-GGT did not show good predictive value for disease diagnosis, with AUCs of 0.572 and 0.647, respectively, both below 0.7. The remaining indicators—TyG-WHtR, TyG-BMI, TyG-WC, METS-IR, and HOMA-IR—all had AUCs greater than 0.7, with TyG-WC having the highest AUC of 0.764, indicating relatively high predictive value for diagnosis.

### 3.5 Construction of predictive model for LF in NAFLD patients and evaluation of its effectiveness

**Figure 5:**
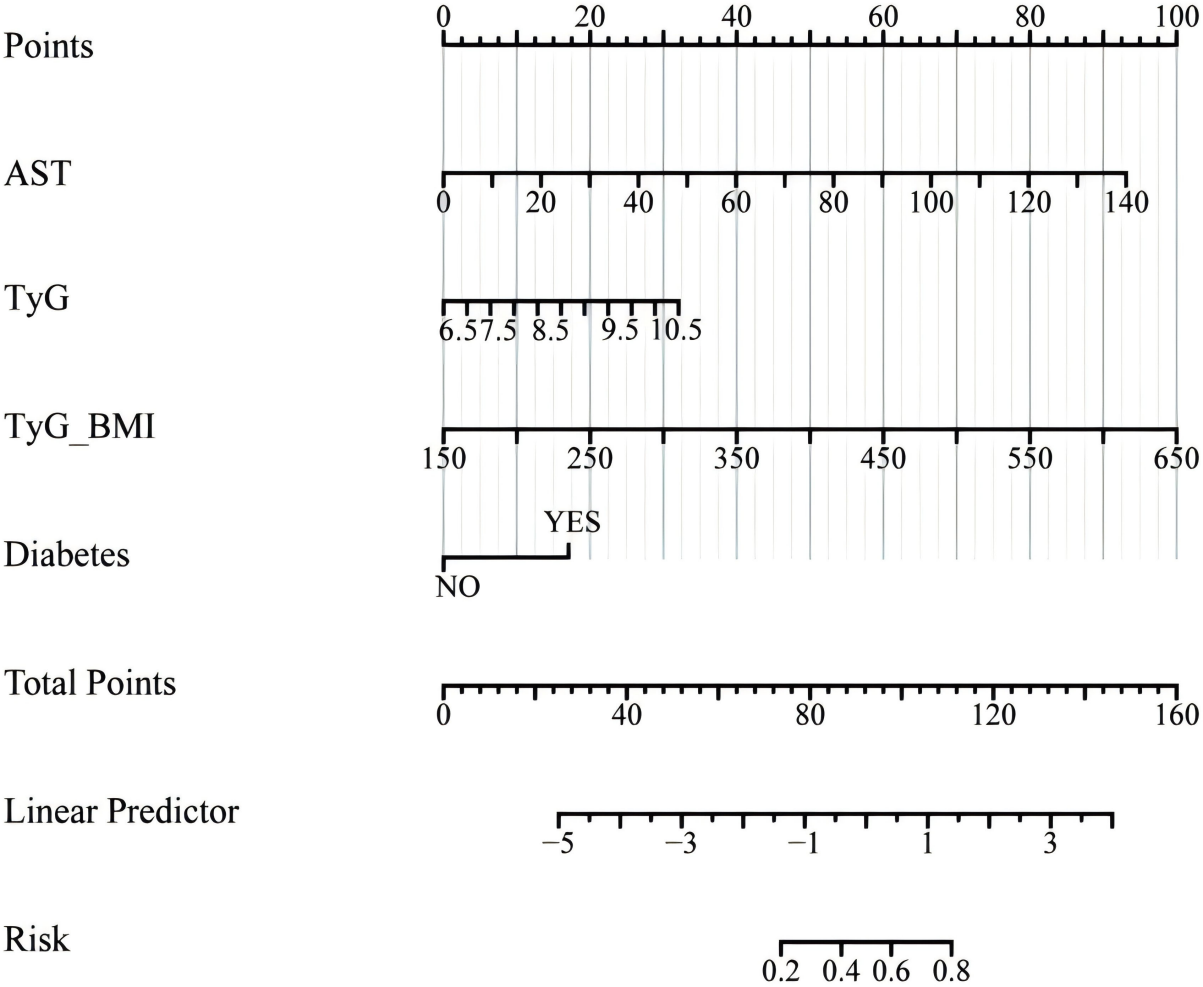
Nomogram for predicting the risk of LF. LF, liver fibrosis.

**Figure 6:**
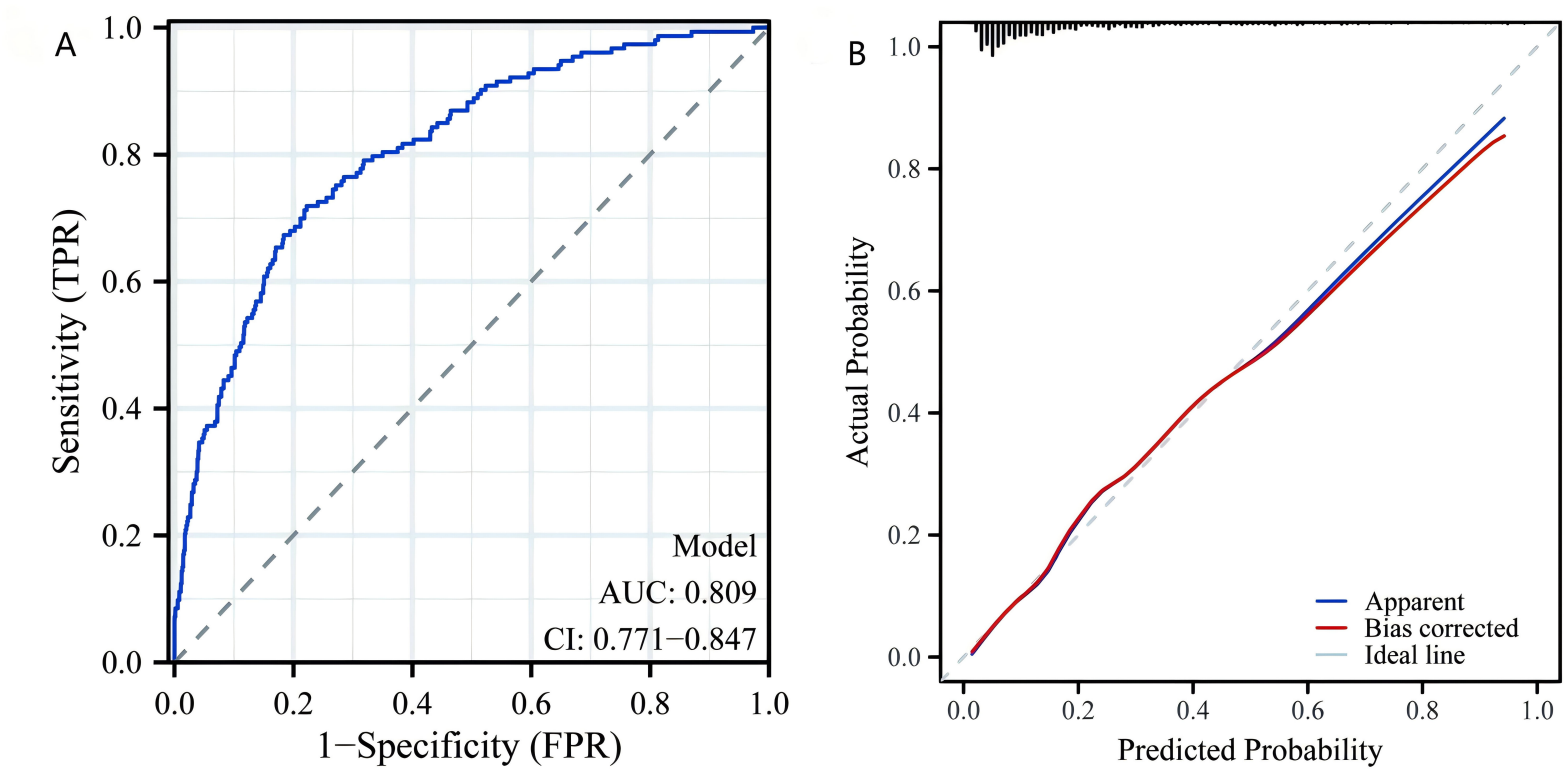
Nomogram model validation. **(A)** Receiver operating characteristic curve for evaluating the discriminative ability of the predictive model. **(B)** Calibration plot for assessing the agreement between predicted probabilities and actual outcomes of LF. LF, liver fibrosis.

We performed a multivariate logistic regression analysis on all indicators to construct the diagnostic model. From 24 variables, we identified four variables as risk factors for predicting LF: AST, TyG, TyG-BMI, and diabetes. The risk scores for each factor included in the nomogram are shown in **Figure 5**, with higher scores indicating a higher risk of LF. To evaluate the performance of the constructed nomogram, we plotted the ROC curve and the calibration curve in **Figure 6**. The ROC curve shows an AUC of 0.809 (95% CI:0.771−0.847), and the calibration curve closely approximates the diagonal, indicating considerable consistency and high calibration quality. These results suggest that the nomogram has good predictive performance.

## 4 Discussion

This study systematically investigated the relationship between various IR indices and LF among patients with NAFLD. Our results demonstrated that indices such as TyG-WHtR, TyG-BMI, and HOMA-IR were significantly associated with the occurrence of LF in NAFLD patients, with TyG-WHtR emerging as the most prominent predictor. Even after adjusting for a range of covariates, TyG-WHtR maintained a strong correlation, suggesting its potential utility as an independent predictor of LF in this patient population. Additionally, we developed a predictive model for LF in NAFLD patients, which highlights the potential of these indices to be incorporated into routine clinical practice for risk assessment and early intervention.

The findings of this study have significant implications for clinical practice, particularly in the early identification and management of LF in patients with NAFLD. The strong association between TyG-WHtR and LF underscores its potential as a simple, non-invasive tool for risk stratification in routine clinical settings. The TyG-WHtR is a non-invasive, simple, low-cost index that only tests TG, FPG, WC, and height to produce results. Compared to liver puncture biopsy, CT, and MRI, TyG-WHtR offers a superior cost-benefit ratio. By incorporating TyG-WHtR and other IR indices into standard metabolic assessments, clinicians can more effectively identify high-risk patients who may benefit from closer monitoring or early intervention. The predictive nomogram developed in this study, which integrates AST, TyG, TyG-BMI, and diabetes, provides a practical and accessible tool for individualized risk assessment. This approach is particularly valuable in resource-limited settings where advanced diagnostic tools may not be readily available. Clinicians can use this nomogram to estimate fibrosis risk using readily available clinical data, enabling targeted therapies such as lifestyle modifications, weight management, and insulin-sensitizing treatments to slow or prevent fibrosis progression (33, 34). These findings advocate for the integration of IR indices into routine clinical practice to enhance early detection, risk assessment, and personalized management of NAFLD-related fibrosis.

Our findings emphasize that obesity, as reflected by higher BMI and WC, and other metabolic factors, such as increased ALT, AST, GGT, and HbA1c levels, were more prevalent in NAFLD patients with LF compared to those without. The liver, an essential organ for metabolic processes, regulates the metabolism of both lipids and glucose. Chiang et al. reported that increased obesity and IR significantly contribute to the progression from NASH to fibrosis through the development of a profibrotic environment in the liver (35). Additionally, Koppe et al. reported that IR leads to widespread metabolic disturbances, resulting in a net effect of TG accumulation in the liver. Some patients may develop hepatocellular injury and LF, which can progress to cirrhosis (36). In comparison to previous research, Khamseh et al. identified TyG-WC, TyG-BMI, and TyG-WHtR as the best predictors of metabolic-associated fatty liver disease (37). Although their study did not establish a clear relationship between these indicators and LF, our research further confirms this link. We found that several IR markers, particularly TyG-WHtR, TyG-BMI, and HOMA-IR, were significantly elevated in LF patients, indicating that metabolic dysfunction plays a central role in the pathogenesis of the disease.

IR indices are not only widely applied in metabolic diseases such as type 2 diabetes and obesity but are also used in other conditions, including cardiovascular diseases, chronic kidney disease, and polycystic ovary syndrome, where they have also been shown to predict adverse outcomes (38–41). Several studies have demonstrated that IR triggers lipotoxic pathways in the liver, leading to an accumulation of toxic lipid species such as ceramides and diacylglycerol, which further exacerbate liver injury and fibrogenesis (42–44). TyG-WHtR showed a significant correlation with LF in this study. High levels of TyG-WHtR indicate severe visceral fat accumulation, which is a key factor in the progression of LF (45). This relationship is particularly significant in the context of NAFLD, where visceral fat plays a crucial role in metabolic dysfunction. The accumulation of visceral fat is linked to various metabolic complications, including increased liver fat content, which can exacerbate liver inflammation and fibrosis (46). Therefore, TyG-WHtR can serve as an independent predictor of LF risk in NAFLD patients, with potential clinical utility.

In recent years, the TyG index has been increasingly applied in liver diseases, especially in predicting the progression of NAFLD, showing a significant association with NAFLD and LF (37, 47). To better evaluate the combined effects of IR and obesity, TyG-BMI integrates BMI, which reflects overall body weight status, making it more advantageous in assessing metabolic risk (22, 48). Our study found that TyG-BMI levels were significantly elevated in LF patients, highlighting the core role of the synergy between obesity and IR in LF progression. Compared to single IR indicators, TyG-BMI provides a more comprehensive assessment of metabolic risk, offering important insights for early identification and intervention of LF. Additionally, in our study, we found that TyG-GGT is not an independent risk factor for LF in NAFLD patients. In contrast, Lei Jin et al. suggested that TyG-GGT has strong predictive accuracy for advanced LF in overweight or obese patients (25). However, their study was limited by a small sample size, a retrospective design that did not fully control for confounding factors, and a lack of strong statistical significance. Additionally, both METS-IR and HOMA-IR are strongly associated with LF, reflecting the relationship between IR and metabolic syndrome. Consistent with previous studies, our findings show that these indicators have strong predictive power for LF in NAFLD patients (49–51). Additionally, HOMA-IR is considered an independent predictor of advanced LF in non-diabetic NAFLD patients (50). It accelerates fibrosis progression by promoting liver fat accumulation, inflammatory responses, and hepatic stellate cell activation (52). Therefore, METS-IR and HOMA-IR can serve as effective tools in clinical practice for assessing the risk of fibrosis in NAFLD patients. In addition to our findings, several studies from China have also reported a strong association between IR and the progression of NAFLD, as well as its correlation with fibrosis staging (47, 53). By integrating these findings, our study contributes to a growing body of evidence that underscores the clinical utility of IR indices in predicting LF risk in NAFLD patients.

This study has several notable strengths. First, it focuses on a specific population of NAFLD patients with LF, making the results more targeted and clinically relevant, thereby providing important insights for the management of this high-risk group. Second, we systematically employed various analytical methods, including logistic regression and RCS, to comprehensively evaluate the relationship between multiple IR indices and LF, clarifying the predictive value of these indices. Finally, we developed a LF prediction model based on multivariable logistic regression and constructed a nomogram, providing a scientific and effective tool for early risk identification and individualized intervention in clinical practice, with high practical value.

Despite providing further evidence of the close relationship between IR and LF in NAFLD patients, our study has several limitations that warrant discussion. First, as this study is based on cross-sectional data, we cannot establish a causal relationship between IR and LF. Longitudinal studies are therefore needed to verify the causal role of IR in the progression of NAFLD. Second, the relatively small sample size and the fact that our cohort was limited to the U.S. population may limit the external validity of the findings, particularly across different ethnicities and regions. Future research should include larger cohorts from diverse populations to validate the applicability and predictive value of these IR indices in a broader context. Additionally, our study mainly focused on epidemiological associations and lacked an in-depth exploration of the underlying molecular mechanisms. Thus, future basic research should aim to elucidate how IR promotes LF through specific cellular signaling pathways, providing theoretical support for targeted interventions.

## 5 Conclusion

In summary, this study identifies TyG-WHtR, TyG-BMI, and other IR indices as independent predictors of LF in NAFLD patients, highlighting their clinical utility in early risk stratification. These findings underscore the importance of integrating metabolic indicators into routine clinical practice to enhance early detection and intervention. The predictive nomogram developed in this study offers a practical, non-invasive tool for clinicians to identify high-risk patients. By focusing on metabolic risk factors, clinicians can implement targeted therapies—such as lifestyle modifications and insulin-sensitizing treatments—to slow or prevent fibrosis progression, ultimately improving long-term patient outcomes.

## Data Availability

The original contributions presented in the study are included in the article/**Supplementary Material**. Further inquiries can be directed to the corresponding author.

## References

[1] Dietrich P Hellerbrand C. Non-alcoholic fatty liver disease, obesity and the metabolic syndrome. Best Pract Res Clin Gastroenterol. (2014) 28:637–53. doi: 10.1016/j.bpg.2014.07.008 25194181

[2] Devarbhavi H Asrani SK Arab JP Nartey YA Pose E Kamath PS. Global burden of liver disease: 2023 update. J Hepatol. (2023) 79:516–37. doi: 10.1016/j.jhep.2023.03.017 36990226

[3] MaChado MV Diehl AM. Pathogenesis of nonalcoholic steatohepatitis. Gastroenterology. (2016) 150:1769–77. doi: 10.1053/j.gastro.2016.02.066 PMC4887389 26928243

[4] Younossi ZM Koenig AB Abdelatif D Fazel Y Henry L Wymer M. Global epidemiology of nonalcoholic fatty liver disease-meta-analytic assessment of prevalence, incidence, and outcomes. Hepatology. (2016) 64:73–84. doi: 10.1002/hep.28431 26707365

[5] Chalasani N Younossi Z Lavine JE Charlton M Cusi K Rinella M. The diagnosis and management of nonalcoholic fatty liver disease: practice guidance from the American association for the study of liver diseases. Hepatology. (2018) 67:328–57. doi: 10.1002/hep.29367 28714183

[6] Perry RJ Samuel VT Petersen KF Shulman GI. The role of hepatic lipids in hepatic insulin resistance and type 2 diabetes. Nature. (2014) 510:84–91. doi: 10.1038/nature13478 24899308 PMC4489847

[7] Shi J Chen J Zhang Z Qian G. Multi-dimensional comparison of abdominal obesity indices and insulin resistance indicators for assessing nafld. BMC Public Health. (2024) 24:2161. doi: 10.1186/s12889-024-19657-6 39123158 PMC11311916

[8] Yetim A Sahin M Kandemir I Bulakci B Aksakal MT Karapinar E. Evaluation of the ability of insulin resistance and lipid-related indices to predict the presence of nafld in obese adolescents. Lipids Health Dis. (2024) 23:208. doi: 10.1186/s12944-024-02144-7 38956572 PMC11218074

[9] Du T Yuan G Zhang M Zhou X Sun X Yu X. Clinical usefulness of lipid ratios, visceral adiposity indicators, and the triglycerides and glucose index as risk markers of insulin resistance. Cardiovasc Diabetol. (2014) 13:146. doi: 10.1186/s12933-014-0146-3 25326814 PMC4209231

[10] Tao LC Xu JN Wang TT Hua F Li JJ. Triglyceride-glucose index as a marker in cardiovascular diseases: landscape and limitations. Cardiovasc Diabetol. (2022) 21:68. doi: 10.1186/s12933-022-01511-x 35524263 PMC9078015

[11] Xue Y Xu J Li M Gao Y. Potential screening indicators for early diagnosis of nafld/mafld and liver fibrosis: triglyceride glucose index-related parameters. Front Endocrinol (Lausanne). (2022) 13:951689. doi: 10.3389/fendo.2022.951689 36120429 PMC9478620

[12] Li S Feng L Ding J Zhou W Yuan T Mao J. Triglyceride glucose-waist circumference: the optimum index to screen nonalcoholic fatty liver disease in non-obese adults. BMC Gastroenterol. (2023) 23:376. doi: 10.1186/s12876-023-03007-8 37919650 PMC10621119

[13] Zou H Ma X Zhang F Xie Y. Comparison of the diagnostic performance of twelve noninvasive scores of metabolic dysfunction-associated fatty liver disease. Lipids Health Dis. (2023) 22:145. doi: 10.1186/s12944-023-01902-3 37674196 PMC10481547

[14] Ajmera V Loomba R. Imaging biomarkers of nafld, nash, and fibrosis. Mol Metab. (2021) 50:101167. doi: 10.1016/j.molmet.2021.101167 33460786 PMC8324681

[15] Tapper EB Loomba R. Noninvasive imaging biomarker assessment of liver fibrosis by elastography in nafld. Nat Rev Gastroenterol Hepatol. (2018) 15:274–82. doi: 10.1038/nrgastro.2018.10 PMC7504909 29463906

[16] Tsochatzis EA Gurusamy KS Ntaoula S Cholongitas E Davidson BR Burroughs AK. Elastography for the diagnosis of severity of fibrosis in chronic liver disease: A meta-analysis of diagnostic accuracy. J Hepatol. (2011) 54:650–9. doi: 10.1016/j.jhep.2010.07.033 21146892

[17] Fan R Yu N Li G Arshad T Liu WY Wong GL. Machine-learning model comprising five clinical indices and liver stiffness measurement can accurately identify masld-related liver fibrosis. Liver Int. (2024) 44:749–59. doi: 10.1111/liv.15818 38131420

[18] Gabriel-Medina P Ferrer-Costa R Ciudin A Augustin S Rivera-Esteban J Pericas JM. Accuracy of a sequential algorithm based on fib-4 and elf to identify high-risk advanced liver fibrosis at the primary care level. Intern Emerg Med. (2024) 19:745–56. doi: 10.1007/s11739-023-03441-2 PMC11039533 37952070

[19] Guo Y Shen B Xue Y Li Y. Development and validation of a non-invasive model for predicting significant fibrosis based on patients with nonalcoholic fatty liver disease in the United States. Front Endocrinol (Lausanne). (2023) 14:1207365. doi: 10.3389/fendo.2023.1207365 37732127 PMC10507901

[20] Xie R Xiao M Li L Ma N Liu M Huang X. Association between sii and hepatic steatosis and liver fibrosis: A population-based study. Front Immunol. (2022) 13:925690. doi: 10.3389/fimmu.2022.925690 36189280 PMC9520084

[21] Guerrero-Romero F Simental-Mendia LE Gonzalez-Ortiz M Martinez-Abundis E Ramos-Zavala MG Hernandez-Gonzalez SO. The product of triglycerides and glucose, a simple measure of insulin sensitivity. Comparison with the euglycemic-hyperinsulinemic clamp. J Clin Endocrinol Metab. (2010) 95:3347–51. doi: 10.1210/jc.2010-0288 20484475

[22] Er LK Wu S Chou HH Hsu LA Teng MS Sun YC. Triglyceride glucose-body mass index is a simple and clinically useful surrogate marker for insulin resistance in nondiabetic individuals. PloS One. (2016) 11:e0149731. doi: 10.1371/journal.pone.0149731 26930652 PMC4773118

[23] Lim J Kim J Koo SH Kwon GC. Comparison of triglyceride glucose index, and related parameters to predict insulin resistance in Korean adults: an analysis of the 2007-2010 Korean national health and nutrition examination survey. PloS One. (2019) 14:e0212963. doi: 10.1371/journal.pone.0212963 30845237 PMC6405083

[24] Lee JH Kwon YJ Park K Lee HS Park HK Han JH. Metabolic score for insulin resistance is inversely related to incident advanced liver fibrosis in patients with non-alcoholic fatty liver disease. Nutrients. (2022) 14(15):3039. doi: 10.3390/nu14153039 35893894 PMC9330359

[25] Jin L Gu J Zhang Z Du CF Xu FQ Huang XK. Tyg-ggt is a reliable non-invasive predictor of advanced liver fibrosis in overweight or obese individuals. Obes Surg. (2024) 34:1333–42. doi: 10.1007/s11695-024-07139-y 38427150

[26] Pang Y Kartsonaki C Turnbull I Guo Y Chen Y Clarke R. Adiposity in relation to risks of fatty liver, cirrhosis and liver cancer: A prospective study of 0.5 million Chinese adults. Sci Rep. (2019) 9:785. doi: 10.1038/s41598-018-36460-7 30692555 PMC6349919

[27] Sun J Yan C Wen J Wang F Wu H Xu F. Association between different obesity patterns and the risk of nafld detected by transient elastography: A cross-sectional study. BMC Gastroenterol. (2024) 24:221. doi: 10.1186/s12876-024-03303-x 38987694 PMC11238456

[28] Sperandei S. Understanding logistic regression analysis. Biochem Med (Zagreb). (2014) 24:12–8. doi: 10.11613/BM.2014.003 PMC3936971 24627710

[29] Arnes JI Hapfelmeier A Horsch A Braaten T. Greedy knot selection algorithm for restricted cubic spline regression. Front Epidemiol. (2023) 3:1283705. doi: 10.3389/fepid.2023.1283705 38455941 PMC10910934

[30] Marrie RA Dawson NV Garland A. Quantile regression and restricted cubic splines are useful for exploring relationships between continuous variables. J Clin Epidemiol. (2009) 62:511–7 e1. doi: 10.1016/j.jclinepi.2008.05.015 19135859

[31] Hanley JA McNeil BJ. The meaning and use of the area under a receiver operating characteristic (Roc) curve. Radiology. (1982) 143:29–36. doi: 10.1148/radiology.143.1.7063747 7063747

[32] Balachandran VP Gonen M Smith JJ DeMatteo RP. Nomograms in oncology: more than meets the eye. Lancet Oncol. (2015) 16:e173–80. doi: 10.1016/S1470-2045(14)71116-7 PMC4465353 25846097

[33] Bischoff M Zimny S Feiner S Sauter J Sydor S Denk G. Multidisciplinary lifestyle intervention is associated with improvements in liver damage and in surrogate scores of nafld and liver fibrosis in morbidly obese patients. Eur J Nutr. (2022) 61:2725–35. doi: 10.1007/s00394-022-02846-7 PMC9279260 35277756

[34] Rakoski MO Singal AG Rogers MA Conjeevaram H. Meta-analysis: insulin sensitizers for the treatment of non-alcoholic steatohepatitis. Aliment Pharmacol Ther. (2010) 32:1211–21. doi: 10.1111/j.1365-2036.2010.04467.x 20955440

[35] Chiang DJ Pritchard MT Nagy LE. Obesity, diabetes mellitus, and liver fibrosis. Am J Physiol Gastrointest Liver Physiol. (2011) 300:G697–702. doi: 10.1152/ajpgi.00426.2010 PMC3094133 21350183

[36] Koppe SW. Obesity and the liver: nonalcoholic fatty liver disease. Transl Res. (2014) 164:312–22. doi: 10.1016/j.trsl.2014.06.008 25028077

[37] Khamseh ME Malek M Jahangiri S Nobarani S Hekmatdoost A Salavatizadeh M. Insulin resistance/sensitivity measures as screening indicators of metabolic-associated fatty liver disease and liver fibrosis. Dig Dis Sci. (2024) 69:1430–43. doi: 10.1007/s10620-024-08309-9 38438774

[38] Ke Z Wen H Huang R Xu X Yang K Liu W. Long-term insulin resistance is associated with frailty, frailty progression, and cardiovascular disease. J Cachexia Sarcopenia Muscle. (2024) 15:1578–86. doi: 10.1002/jcsm.13516 PMC11294012 39031905

[39] Lee JH Jeon S Joung B Lee HS Kwon YJ. Associations of homeostatic model assessment for insulin resistance trajectories with cardiovascular disease incidence and mortality. Arterioscler Thromb Vasc Biol. (2023) 43:1719–28. doi: 10.1161/ATVBAHA.123.319200 37470180

[40] Diamanti-Kandarakis E Dunaif A. Insulin resistance and the polycystic ovary syndrome revisited: an update on mechanisms and implications. Endocr Rev. (2012) 33:981–1030. doi: 10.1210/er.2011-1034 23065822 PMC5393155

[41] Lin CA Li WC Lin SY Chen YC Yu W Huang HY. Gender differences in the association between insulin resistance and chronic kidney disease in a Chinese population with metabolic syndrome. Diabetol Metab Syndr. (2022) 14:184. doi: 10.1186/s13098-022-00956-0 36461016 PMC9716739

[42] Samuel VT Shulman GI. Mechanisms for insulin resistance: common threads and missing links. Cell. (2012) 148:852–71. doi: 10.1016/j.cell.2012.02.017 PMC3294420 22385956

[43] Wang K Wei Y Xu R Li Y Mao C. Manifold roles of ceramide metabolism in non-alcoholic fatty liver disease and liver cancer. Adv Exp Med Biol. (2022) 1372:157–68. doi: 10.1007/978-981-19-0394-6_11 35503180

[44] Yu A Cable C Sharma S Shihan MH Mattis AN Mileva I. Targeting acid ceramidase ameliorates fibrosis in mouse models of non-alcoholic steatohepatitis. Front Med (Lausanne). (2022) 9:881848. doi: 10.3389/fmed.2022.881848 36275798 PMC9582277

[45] Zhang X Wang Y Li Y Gui J Mei Y Yang X. Optimal obesity- and lipid-related indices for predicting type 2 diabetes in middle-aged and elderly Chinese. Sci Rep. (2024) 14:10901. doi: 10.1038/s41598-024-61592-4 38740846 PMC11091178

[46] Kumar A Arora A Sharma P Jan S Ara I. Visceral fat and diabetes: associations with liver fibrosis in metabolic dysfunction-associated steatotic liver disease. J Clin Exp Hepatol. (2025) 15:102378. doi: 10.1016/j.jceh.2024.102378 39268479 PMC11387673

[47] Zhang F Han Y Wu Y Bao Z Zheng G Liu J. Association between triglyceride glucose-body mass index and the staging of non-alcoholic steatohepatitis and fibrosis in patients with non-alcoholic fatty liver disease. Ann Med. (2024) 56:2409342. doi: 10.1080/07853890.2024.2409342 39348274 PMC11443541

[48] Gui J Li Y Liu H Guo LL Li J Lei Y. Obesity- and lipid-related indices as a predictor of obesity metabolic syndrome in a national cohort study. Front Public Health. (2023) 11:1073824. doi: 10.3389/fpubh.2023.1073824 36875382 PMC9980350

[49] Kuo TC Lu YB Yang CL Wang B Chen LX Su CP. Association of insulin resistance indicators with hepatic steatosis and fibrosis in patients with metabolic syndrome. BMC Gastroenterol. (2024) 24:26. doi: 10.1186/s12876-023-03095-6 38195414 PMC10775571

[50] Koo DJ Lee MY Jung I Moon SJ Kwon H Park SE. Baseline homeostasis model assessment of insulin resistance associated with fibrosis progression in patients with nonalcoholic fatty liver disease without diabetes: A cohort study. PloS One. (2021) 16:e0255535. doi: 10.1371/journal.pone.0255535 34432804 PMC8386882

[51] Ramirez-Manent JI Martinez-Almoyna E Lopez C Busquets-Cortes C Gonzalez San Miguel H Lopez-Gonzalez AA. Relationship between insulin resistance risk scales and non-alcoholic fatty liver disease and liver fibrosis scales in 219,477 Spanish workers. Metabolites. (2022) 12:1093. doi: 10.3390/metabo12111093 36355176 PMC9698315

[52] Fujii H Imajo K Yoneda M Nakahara T Hyogo H Takahashi H. Homa-ir: an independent predictor of advanced liver fibrosis in nondiabetic non-alcoholic fatty liver disease. J Gastroenterol Hepatol. (2019) 34:1390–5. doi: 10.1111/jgh.14595 30600551

[53] Yang RX Zou ZS Zhong BH Deng H He FP Shi JP. The pathologic relevance of metabolic criteria in patients with biopsy-proven nonalcoholic fatty liver disease and metabolic dysfunction associated fatty liver disease: A multicenter cross-sectional study in China. Hepatobiliary Pancreat Dis Int. (2021) 20:426–32. doi: 10.1016/j.hbpd.2021.06.002 34246549

